# Silicon Multi-Pass Gas Cell for Chip-Scale Gas Analysis by Absorption Spectroscopy

**DOI:** 10.3390/mi11050463

**Published:** 2020-04-28

**Authors:** Alaa Fathy, Yasser M. Sabry, Martine Gnambodoe-Capochichi, Frederic Marty, Diaa Khalil, Tarik Bourouina

**Affiliations:** 1 ESYCOM Lab, UMR 9007 CNRS, Université Gustave Eiffel, ESIEE Paris, 77454 Marne-la-Vallée, France; alaa.fathy@esiee.fr (A.F.); martine.capo-chichi@u-pem.fr (M.G.-C.); frederic.marty@esiee.fr (F.M.); 2 Si-Ware Systems, 3 Khalid Ibn Al-Waleed St., Heliopolis, Cairo 11361, Egypt; yasser.sabry@eng.asu.edu.eg (Y.M.S.); diaa_khalil@eng.asu.edu.eg (D.K.); 3 Faculty of Engineering, Ain-Shams University, 1 Elsarayat St. Abbassia, Cairo 11517, Egypt

**Keywords:** circular multi-pass cell, silicon integrated, gas sensing

## Abstract

Semiconductor and micro-electromechanical system (MEMS) technologies have been already proved as strong solutions for producing miniaturized optical spectrometers, light sources and photodetectors. However, the implementation of optical absorption spectroscopy for in-situ gas analysis requires further integration of a gas cell using the same technologies towards full integration of a complete gas analysis system-on-chip. Here, we propose design guidelines and experimental validation of a gas cell fabricated using MEMS technology. The architecture is based on a circular multi-pass gas cell in a miniaturized form. Simulation results based on the proposed modeling scheme helps in determining the optimum dimensions of the gas cell, given the constraints of micro-fabrication. The carbon dioxide spectral signature is successfully measured using the proposed integrated multi-pass gas cell coupled with a MEMS-based spectrometer.

## 1. Introduction

Gas sensing is gaining increased interest mainly for air pollution monitoring and to trigger air purification systems when needed. Gas sensors are used in multiple industrial applications for process control and predictive maintenance [1]. They are considered as key devices in emerging Internet-of-Things (IoT) applications, including monitoring food freshness [2]. The massive deployment of gas sensors is also being considered in cars [3], buildings and even for integration into smartphones. Sensor miniaturization is dominated nowadays by semiconductor technologies, including micro-electromechanical system (MEMS) mainly due to their potential for volume manufacturing at low cost. These technologies are suitable mostly for physical sensors, and to a lesser extent, some chemical sensors, including miniaturized analytical chemistry systems such as gas chromatography [4] and optical spectroscopy [5,6,7,8]. While chromatography requires collection and injection of a sample of the analyte into a chromatographic column, optical spectroscopy offers the great advantage of allowing non-invasive analysis thanks to light–matter interaction.

When considering optical absorption spectroscopy, it is of high interest to use infrared light when dealing with gases, either in Near-Infra-Red (NIR) or, even more interestingly, in Mid-Infra-Red (MIR), where the absorptivity ε(λ) is higher, leading to more intense absorption lines in this particular spectral range [9]. Furthermore, efficient gas absorption of light also requires a long-enough interaction length *L*, according to the Beer–Lambert law: $A_{p}=P_{0}(1-exp\left[c.\varepsilon \left(\lambda \right).L\right]$), where $A_{p}$ is the amount of absorbed light, $P_{0}$ is the incident light, c the gas concentration and ε(λ) its wavelength-dependent absorptivity. Practically, this light–matter interaction occurs inside dedicated gas cells, which are key components, enabling good control of both the sample being measured and the optical path *L*.

Multi-pass cells are famous in gas sensing for increasing the sensitivity by bouncing light in a long path within a small volume using high reflectivity mirrors. The larger path length to cell volume ratio means a smaller gas cell which leads to a compact gas sensor. The more interaction with the light without introducing losses, the better the sensitivity of the gas sensor.

Multi-pass cells also have different applications [10] such as optical delay line, a non-linear tool for a spectral broadening of laser pulses [11,12] and are used in magnetometers [13].

In fact, the design of gas cells benefits from a rich research background. Gas cells’ classification includes White [14], Herriott [10], an astigmatic Herriott [15], a multi-pass matrix system [16], circular gas cells [17] and an integrating sphere [18]. Circular multi-pass cells can be implemented using flat concentric mirrors (mirror for each reflection) [19], spherical concentric mirrors (light hits each mirror more than once) [20], a single toroidal mirror (different radius of curvature in the sagittal and tangential plane to correct astigmatism aberration) [21] or circular cell of multiple rows in the vertical direction [22].

However, when dealing with such gas cells at the chip scale, as considered in our work, we are facing two major difficulties related to miniaturization. First, the lateral dimensions of a chip are limited to a few millimeters, which is a severe limitation to the optical path *L*, hence reducing the light–gas interaction. One can deal with this first limitation by adopting the concept of multi-pass gas cells. Second, one also has to accommodate other optical considerations related to the miniaturization of the gas cells. For instance, we need to make sure that optical loss is mainly governed by gas absorption, hence the need for careful optical design to minimize the insertion loss. The loss can be significantly affected by the small thickness of silicon wafers, which is limited to no more than a few hundred micrometers. Such small thickness hinders the optical throughput of the cell operating with free-space propagating light as it makes it difficult to collimate an incident light of small spot-size with large throughput into the silicon chip. On the other side, the use of the lithographic process offers the great advantage of high-quality self-alignment of the micro-fabricated optical parts according to a given computer-aided design (CAD) mask design. This can lead to high mechanical stability as well since all components are micromachined in the same substrate. This allows implementing sophisticated designs without the need for post-fabrication alignment.

In this work, we present a circular gas cell micro-fabricated on a silicon substrate using optical MEMS technology, where all cell elements including the coupling mirrors are implemented on the same chip. Design methodology and models are derived leading to guidelines and important considerations. The proposed cell is fabricated and preliminary experimental results are introduced. Carbon dioxide gas is measured using the proposed cell together with a MEMS-based Fourier Transform Infra-Red Spectrometer (FTIR) as a proof of concept.

## 2. Materials and Methods

### 2.1. Design Considerations

**Figure 1:**
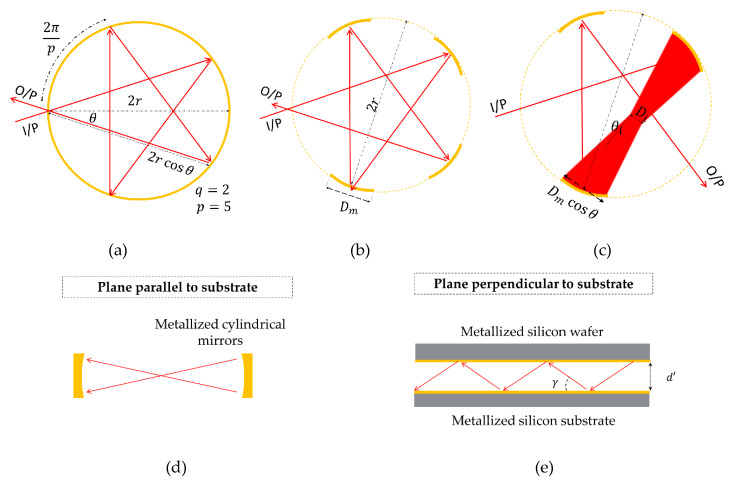
Schematic top views of circular multi-pass gas cells of radius R. (**a**) Conventional. (**b**) Using cylindrical mirrors instead of a one-piece circular mirror. (**c**) Modified with cylindrical mirrors, where the last mirror of the light path is omitted. Red rays represent the central light path. The extended beam is only drawn here to avoid figure complexity. (**d**) Configurations for light guiding in integrated gas cells. The drawing illustrates guiding light in the plane parallel to the substrate where cylindrical mirrors are used. (**e**) The drawing shows a side view illustrating an alternative method for guiding light in the plane perpendicular to the substrate using the horizontal metalized silicon substrate and the metalized capping (silicon wafer). *d* denotes the cell height and γ is the angle between the ray and the substrate.

An example of the conventional circular multi-pass cell is shown in Figure 1a. The light path (red rays) forms a star polygon [23]. The star polygon is characterized by a radius r, the number of vertices p lying on the circle circumstance and density of the star q. Each segment is connected from a vertex to another one spaced q positions. The input tilt angle is given by $\theta =\frac{\pi }{2}\left(1-\frac{2q}{p}\right).$ In our work, cylindrical mirrors can be placed at the positions of the vertices as shown in Figure 1b. These mirrors can improve cell insertion loss and coupling efficiency by tuning its radius of curvature and even by using an acylindrical profile. This can be also used to reduce the aberrations due to multiple reflections inside the cell. In both designs, the circular multi-pass cell suffers from complexity in light coupling because the input and output are on the same side with an angle of 2θ. To mitigate such a limitation, the last mirror impinged by rays in Figure 1b can be omitted, as shown in Figure 1c. This will reduce the total path length by 1/p, which can be neglected for large values of p. Light-guiding/focusing in the plane parallel to the substrate is achieved using cylindrical mirrors (inside the cell), as depicted in Figure 1d. In the proposed configuration and the plane perpendicular to the substrate, light is guided using the metalized substrate and a metalized capping wafer, as depicted in Figure 1e.

### 2.2. Proposed Micro-Fabricated Gas Cells

**Figure 2:**
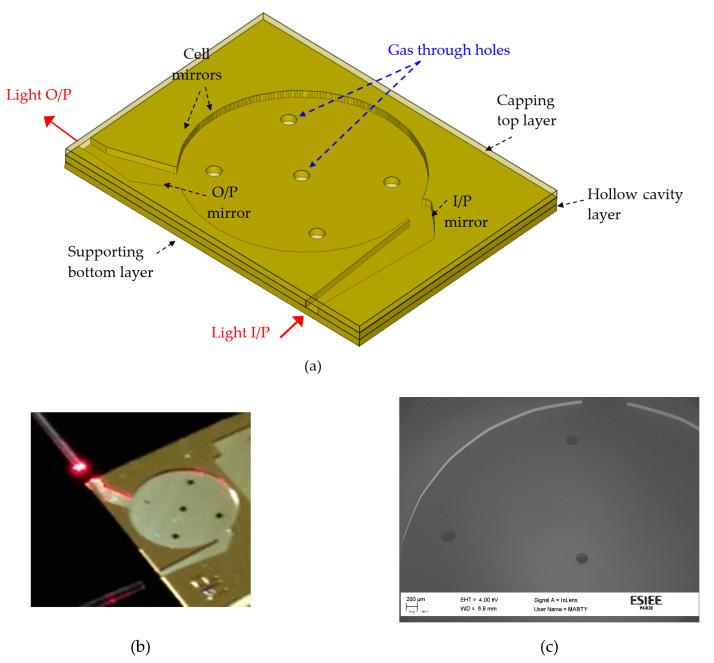
(**a**) Three-dimensional (3D) layout of a silicon integrated gas cell. The device layer contains the gas cell. The substrate contains the gas through holes. Capping layer for covering the cell; (**b**) photo of the fabricated device with optical fiber light coupling in and out; (**c**) close view scanning electron microscope image.

A 3D layout of the proposed gas cell is depicted in Figure 2. The input and output light directions are perpendicular to each other for easy light coupling. A cylindrical input mirror is used to couple the light from the source to inside the cell. The mirror images the light from a width of d to a width $D_{i}.$ The opposite is carried out in the output by another cylindrical mirror. The cell itself comprises cylindrical mirrors which are concentric. The mirrors are self-aligned, where they are micromachined in the same silicon block which is attached to a substrate. The substrate contains the gas through holes as well as optical the input/output. The capping is metalized with another silicon wafer. Gas through holes in the substrate for gas injection into the cell.

### 2.3. Fabrication Steps

The silicon wafer is first thermally oxidized to form a 2 µm thick SiO_2_ layer which serves as a hard mask for the deep reactive ion etching (DRIE) process. The whole-cell design was then transferred to the oxide layer by using a photolithography process followed by a plasma etching of the hard mask. On the backside of the wafer, a second mask was used to create the gas injection holes into the SiO_2_ layer. The next step consists of a deep silicon etching using the Bosch process in an etch tool (Alcatel A601E, Alcatel, Annecy, France). The etching process used a time-multiplexed plasma etch including an etch step with SF_6_ gas for 5 s and a passivation step with C_4_F_8_ gas for 2 s. The etching process was carried out to achieve an etch depth of 200 µm hence shaping the gas cell profile. Another DRIE process was performed from the backside of the wafer to form the gas through holes. These holes are distributed uniformly in the cell for homogenous gas filling and are important for the fast filling of the cell. The hole diameter is 0.4 mm. A smoothing process was then used to lower the surface roughness of the walls and mirrors that originated from the DRIE and, thus, reduced the reflection losses. This smoothing process was carried out by oxidizing the silicon wafer. A 2 µm thick oxide was thermally grown and then removed with wet etching in hydrofluoric acid (HF). Another silicon wafer was used as the capping for the structure. A final sputtering step was used to metalize the cap wafer, as well as the whole gas cells’ wafer, with a 30 nm thick TiW layer covered by a 500 nm thick gold layer. A scanning electron microscope (SEM) image for the cell and a camera photo without the capping are shown in Figure 2b,c, respectively. The cell was illuminated with a red laser from a fiber. Shiny spots at some mirrors are observed. These are the mirrors that are expected to be hit by a laser once entering the gas cell while the other mirrors are not shiny due to light diffraction from the upper side due to the absence of the capping.

### 2.4. Experimental Setup

**Figure 3:**
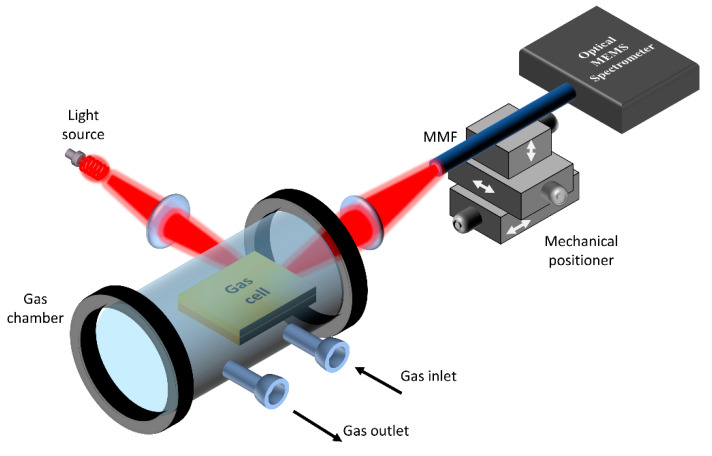
Schematic 3D view of the optical setup used for conducting gas sensing measurements using the silicon integrated multi-pass cell.

To perform gas sensing measurements, an optical setup was used, as depicted in Figure 3. The gas cell was placed inside a macro gas chamber (Storm™10, Specac, Orpington, England). The macro gas cell was used to control gas pressure and concentration. Light from a filament-based light source was coupled to the cell input using a lens. The output from the cell is coupled using another lens to a multimode fiber MMF (Thorlabs, Newton, NJ, USA core/cladding 400/440 μm, NA=0.39). The MMF was connected to the optical MEMS spectrometer (Neospectra, Si-ware Systems, Cairo, Egypt). The resolution of the spectrometer was down to 33 ${\mathrm{cm}}^{-1}$ and its wavelength range extended from 1.2 μm to 2.5 μm.

## 3. Results

### 3.1. Modeling and Simulation

In this section, we are going to find the cell throughput leading us to deduce the absorbed power by the gas as a function of cell parameters. Such an equation will be optimized to find the optimum cell parameters given technology constraints. From an optical point-of-view, the system throughput is limited by the input/output aperture size and numerical aperture (NA). Thus, to avoid additional losses inside the gas cell, the latter should be designed to maintain the throughput without truncation. First, we take into effect the losses in the in-plane where the throughput in such a plane is defined as:

(1) $$TP=d\;NA=D_{i}\theta_{i}$$

where d is the width of the optical aperture, $D_{i}$ is the image width, NA is the device numerical aperture and $\theta_{i}$ is the image divergence angle. The design idea depends on using a cylindrical mirror, which performs 1:1 imaging, as depicted in Figure 1c. The source image is halfway between the two opposite mirrors. Thus, the cylindrical mirrors’ focal length $f_{m}$ is one-quarter the length between two successive mirrors, which is equal to 2rcosθ. As depicted in Figure 1c, the divergence angle of the image is given by:

(2) $$\theta_{i}=\frac{D_{m}\cos\theta -D_{i}}{2r\cos\theta }$$

where $D_{i}<D_{m}.$ In view of Equations (1) and (2), one can find $D_{i}=0.5\left(D_{m}\cos\theta -\sqrt{{\left(D_{m}\cos\theta \right)}^{2}-8\;D_{s}NA_{s}\;r\cos\theta \;}\right).$ This implies that the sum of the terms under the square root to be positive. Knowing that $D_{m}=\frac{2\pi R}{p}$, then one could get the following condition:

(3) $$p^{-2}\sin\pi \frac{q}{p}\geq \frac{2}{\pi^{2}}\frac{d}{r}\gamma_{d}$$

The absorbed power by the gas is given by $A_{P}=P_{out}\left(1-e^{-\alpha L_{T}}\right)$, where $P_{out}$ is the output power from the cell, α is the gas absorption coefficient and $L_{T}$ is the total cell path length and it is equal to (p−1)2rcosθ. For a cell with invariant throughput, $P_{out}=R^{p}P_{in}$, where R is the mirror reflectivity ((p − 2) mirrors are inside the cell and 2 mirrors for the input and output coupling). It can be shown that it is a function of the star polygon parameter p and q. For small gas concentrations ($\alpha L_{T}\ll 1)$, the absorbed power is given by:

(4) $$A_{P}\left(p,q\right)\propto \left(p-1\right)\sin\frac{\pi q}{p}R^{p}$$

Superior performance is achieved when $A_{P}$ is maximum. However, one should take into consideration the following characteristics of the star polygon:

- q and p are integers.
- q/p is relatively prime (co-prime).
- q<0.5p
- q>1

Thus, maximizing Equation (4) given the above conditions in addition to Equation (3), one could find the optimum values of p and q that specify the corresponding $L_{tot}$ and θ. It is also apparent that these values are dependent on the ratio between TP=d NA and r. The optimum values of q and p and the corresponding $L_{T}/r$ is given in Table 1 for different reflectivity R and NA d/r. The longer path length can be achieved for the highest reflectivity and the smaller source throughput to the cell radius. Thus, given a detection system of throughput TP, cell volume (consequently radius r) and technological mirror reflectivity, one can use the table to find the parameters of the optimum design.

The analysis assumes that the reflection losses due to the substrate and capping are negligible. This is the case when the gas cell has a large height, where the light can be easily collimated in the out-of-plane direction or the case of the light with a small divergence angle. In the case of the MEMS chip, the height is limited to hundreds of μm and the light divergence angle is not negligible. In such a case, one should take into account the reflectivity losses in the out-of-plane. For simplicity, one can assume that the two directions are independent. The number of reflections in the out-of-plane (the structure acts as a parallel plate multimode waveguide in such a plane) is dependent on the ray angle. It is given by $L_{T}/\left(d^{\prime }/\tan\gamma \right),\;\gamma$ is the ray angle with respect to the longitudinal direction and $\left(d^{\prime }/\tan\gamma \right)$ is the longitudinal distance between two successive reflections, as defined in Figure 1e. Thus, the output power should be modified to be:

(5) $$P_{out}\propto R{\left(\theta \right)}^{p}\int_{0}^{\frac{\pi }{2}}I\left(\gamma \right)\;R{\left(90-\gamma \right)}^{\frac{L_{T}\tan\gamma }{d^{\prime }}}d\gamma$$

where I(γ) is the radiation intensity of input light, R(90−γ) represents the reflection coefficient in out-of-plane with an incidence angle of (90−γ) and R(θ) represents the reflection coefficient on the cylindrical mirrors with an incidence angle of θ. Then, the percentage of the absorbed power is given by:

(6) $$A_{P}\left(p,q\right)\propto \left(p-1\right)\sin\frac{\pi q}{p}R{\left(\theta \right)}^{p}\;\int_{0}^{\frac{\pi }{2}}I\left(\gamma \right)\;R{\left(90-\gamma \right)}^{2\left(p-1\right)\sin\frac{\pi q}{p}\tan\gamma \;{\left(\frac{d^{\prime }}{r}\right)}^{-1}}d\gamma$$

**Figure 4:**
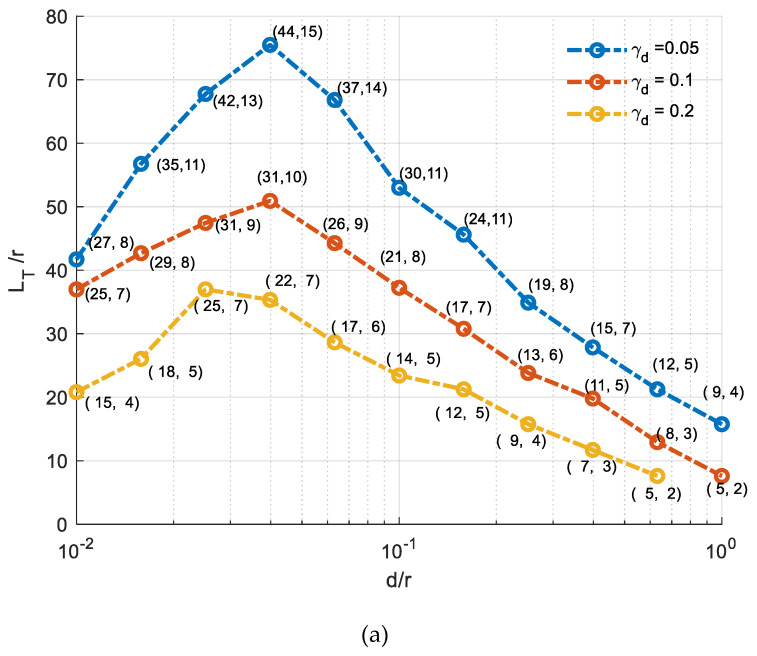

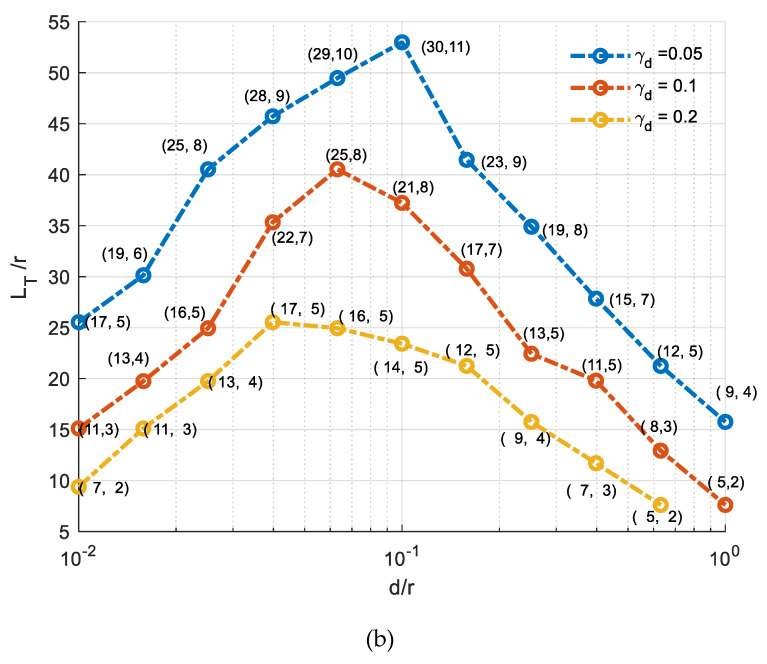
Total path length with respect to cell radius $L_{T}/r$ versus cell height/depth with respect to cell radius d/r for different divergence angle $\gamma_{d}$ in the case of (**a**) gold metallization. (**b**) Aluminum metallization. Text at every point represents (p,q ).

On maximizing the above equation, it is found that the optimum p and q are dependent on the ratio $d^{\prime }/r$, out-of-plane divergence angle $\gamma_{d}$ and metallization reflectivity R. For gold metallization and a square input aperture ($d=d^{\prime })$, $L_{T}/r$ is plotted versus d/r in Figure 4a for different divergence angle at a wavelength of operation of 2 μm. Another set of design curves are plotted for aluminum metallization in Figure 4b. The intensity distribution is assumed Gaussian distribution of full width at half maximum (FWHM) of $\gamma_{d}.$ There is an optimum cell height (or depth) with respect to its radius, which corresponds to a maximum total path length with respect to the cell radius. Such a length is longer for a smaller divergence angle. Using gold metallization, longer path length can be achieved by about 30% over what can be achieved using the aluminum metallization at a wavelength of 2 μm. Knowing the cell sizes (d and r) and metallization material, the corresponding p, q, θ and total path length $L_{T}$ can be deduced for a given design.

### 3.2. Experimental Validation

**Figure 5:**
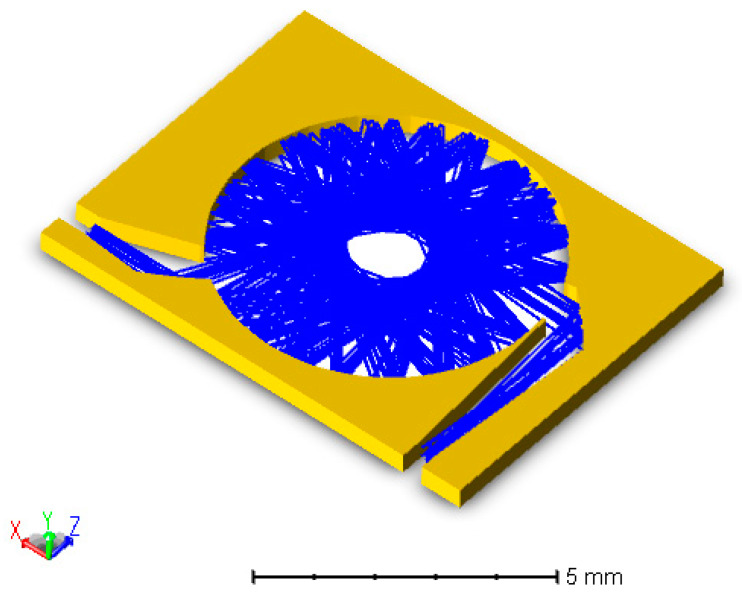
Ray tracing for the integrated multi-pass using ZEMAX.

Optimized gas cells with gold metallization were targeted for fabrication and further characterization. The corresponding target depth *d* is around 190 μm, while the cell radius *r* is in the vicinity of 3 mm. The corresponding d/r ratio is around 0.06, close to maximum sensitivity according to the optimal ratio for $L_{T}/r$ as shown in Figure 4a. After completion of device fabrication, the characterization of the resulting gas cell was carried out using a MEMS FTIR spectrometer. According to the above-mentioned analysis, the specifications of the fabricated cell are calculated and can be found in Table 2. The radius of the curvature of the cylindrical mirrors was optimized using the ray tracing simulator ZEMAX. Ray tracing for the cell is shown in Figure 5.

**Figure 6:**
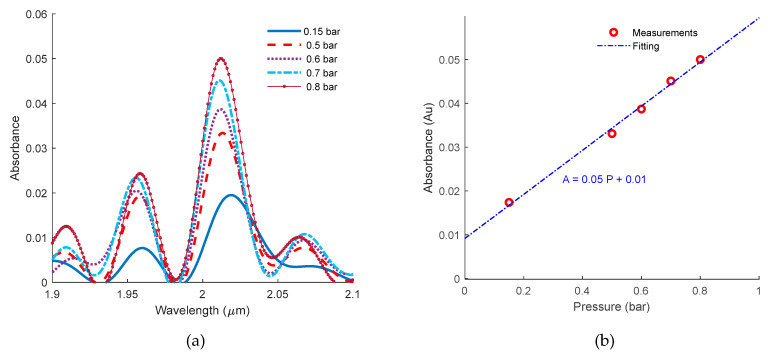
Carbon dioxide measurements. (**a**) Carbon dioxide (CO_2_) absorbance at different pressures measured using the integrated multi-pass cell. (**b**) The corresponding absorbance at 2.01 μm versus different pressures. A linear fitting is also plotted.

Carbon dioxide (CO_2_) gas is injected into the chamber inlet from a gas cylinder. The pressure was adjusted using a pressure valve and monitored using an analog pressure gauge. Different pressures were measured which corresponds to different absorbance values (0.9 bar to 1.6 bar). The corresponding absorbance curves are plotted in Figure 6a. The absorbance values at 2.01 μm versus the pressure applied are scattered in Figure 6b in addition to a linear fit with a root mean square error of $10^{-3}.$

## 4. Discussion

Implementing the gas cell on silicon enables enabled sophisticated designs with no need for further optical alignment, thanks to the lithography-defined geometric features allowing self-alignment capabilities. One can further implement a design incorporating a large number of mirrors of different radii of curvature and acylindrical profiles. In addition to that, the mechanical stability is high as all components are monolithically integrated on the same substrate. However, MEMS technology does not provide a straightforward solution for the out-of-plane focusing [24]. This problem was solved by guiding light using the metalized capping and the metalized substrate acting as a multimode parallel plate waveguide.

The gas cell was used in conjunction with the MEMS spectrometer to measure carbon dioxide at different pressures. An effective path length was found to be 9 cm. The cell volume is too small. It is about 6 μL. This means a small sampling volume of gases. The path length to the volume ratio PVR is an important Merit factor for the gas cells [25], the more this value the more compact the cell you have. The corresponding PVR is about 17,000 m/L. Such value is two orders of magnitudes bigger than that of the conventional gas cells (macro gas cells have PVR values ranging from tens to hundreds, according to the survey conducted in the reference [25]). However, this is at the expense of sensitivity where the benchtop gas cells have larger throughput and lower losses (a smaller number of reflections).

## 5. Conclusions

A new multi-pass gas cell was implemented on-chip using MEMS technologies. Design methodology and guidelines for the optimal design are derived and presented. These results show the optimum dimensions of the gas cell (optimum sensitivity) given technology parameters such as reflectivity and the depth to be achieved besides the spectrometer throughput. The cell in conjunction with a MEMS-based spectrometer was successfully used to measure carbon dioxide with different pressures.

## Figures and Tables

**Table 1:** Optimum values of $\left(q,p,\;L_{T}/R\right)$ for different mirror reflectivity and TP/R.

	$NA\frac{d}{r}\;(\mathrm{rad}.)$	0.01	0.03	0.05	0.07	0.09
R						
0.91		(5, 11, 19.8)	(5, 11, 19.8)	(4, 9, 15.8)	(3, 7, 11.7)	(3, 7, 11.7)
0.93		(6, 13, 23.8)	(5, 11, 19.8)	(4, 9, 15.8)	(3, 8, 12.9)	(3, 7, 11.7)
0.95		(9, 19, 35.9)	(5, 12, 21.3)	(4, 9, 15.8)	(3, 8, 12.9)	(3, 7, 11.7)
0.97		(10, 21, 39.9)	(5, 12, 21.3)	(4, 9, 15.8)	(3, 8, 12.9)	(3, 7, 11.7)
0.99		(10, 21, 39.9)	(5, 12, 21.3)	(4, 9, 15.8)	(3, 8, 12.9)	(3, 7, 11.7)

**Table 2:** Simulated dimensions of the cell. All dimensions are in mm. r is the mirror radius of curvature, and D is the mirror diameter.

Parameter	Value
**Metallization material**	Gold
**Cell radius**	3
**Cell (** p,q **)**	22, 9
**Depth**	0.19
**Input mirror (** $r_{in}$ **,** $D_{in},$ **)**	8.8, 1.2
**Output mirror (** $r_{out},\;D_{out},$ **)**	6.7, 1.4
**Cell mirrors (** $r_{m},\;D_{m}$ **)**	5.3, 0.9
**Cell area**	7 × 8
